# The feasibility and safety of robotic‐assisted salvage radical cystectomy

**DOI:** 10.1002/bco2.459

**Published:** 2024-12-25

**Authors:** Gal Rinott Mizrahi, Nathan Lawrentschuk, Benjamin Thomas, Philip Dundee

**Affiliations:** ^1^ The Royal Melbourne Hospital Parkville Victoria Australia; ^2^ Epworth Hospitals Melbourne Victoria Australia; ^3^ St Vincent's Private Hospital Melbourne Melbourne Victoria Australia

**Keywords:** intraoperative complications, pelvic radiation, postoperative complications, radical cystectomy, robotic‐assisted salvage radical cystectomy (RA‐SRC), salvage

## Abstract

**Objectives:**

To evaluate the feasibility and safety of robotic‐assisted salvage radical cystectomy (RA‐SRC).

**Materials and Methods:**

We retrospectively searched the prospectively collected surgical database of two highly experienced robotic urological surgeons for cases of RA‐SRC, defined as RARC performed post‐previous pelvic RT for palliative or oncologic treatment purposes. Collected data included demographic and clinical information and outcome measures including operative course, hospital stay and complications.

**Results:**

Eighteen patients were included in the current analysis. All patients had previous RT to the pelvis with 12 patients also having prior radical pelvic surgery. Indications for salvage cystectomy were either palliation (*n* = 12) or oncological (MIBC or high risk NMIBC, *n* = 6). There were no intraoperative complications and no conversions to open surgery. Ninety day postoperative complications were recorded in 11 patients (61.1%), with major complications (Clavien–Dindo grades 3 and 4) in three patients (16.6%). After a median follow‐up of 43.5 months, one late postoperative complication was observed requiring surgical intervention.

**Conclusion:**

Our data, together with the limited published data from other cohorts of RA‐SRC, suggest that in experienced hands, RA‐SRC is feasible, with intraoperative and perioperative complication rates that are lower than the published data for open SRC and are equivalent to open primary RC. These data will contribute to treatment decision making both in patients with post‐pelvic radiation symptoms requiring palliation and patients with MIBC considering or treated with trimodal treatment.

## 1 INTRODUCTION

Salvage radical cystectomy (SRC) describes radical cystectomy (RC) following prior unsuccessful trimodal treatment (TMT) for muscle invasive bladder cancer (MIBC) or when RC is performed for purely palliative purposes to treat previous treatment‐related bladder complications, mainly post‐pelvic radiation treatment (RT).^1^ Improvements in surgical and anaesthetic techniques in recent years have reduced the morbidity and mortality associated with RC. However, RC is still considered a major surgery with up to 42% risk of developing high‐grade complications and up to 8% mortality rate.^2^, ^3^

RT is known to induce severe tissue damage that extends beyond the target organ to the surrounding tissue resulting in a desmoplastic reaction. This significantly impairs identification and dissection of tissue planes during surgery. Furthermore, RT increases the risk for impaired tissue repair potentially affecting bowel anastomosis, ureteric anastomosis and wound healing.^4^, ^5^ The distinction of SRC from standard RC is important as it implies increased technical difficulty which may result in higher morbidity and mortality rates and requires enhanced surgical skills.^6^

Different surgical approaches for performing RC are in use, with robotic‐assisted radical cystectomy (RARC) being performed with increasing frequency. Numerous clinical trials and registry‐data analyses published in recent years have revealed improved perioperative outcomes following RARC compared to open RC. Outcomes have included lower intraoperative blood loss, transfusion rates and thromboembolic events, as well as reduced wound and GI complications and shorter length of hospitalization.^7^, ^8^ Given the prevalence of co‐existing illnesses in patients undergoing RC, this population may benefit more than others from robot‐assisted surgery.

There is a paucity of literature describing feasibility and safety of robotic‐assisted SRC (RA‐SRC). In this study, we present our experience with RA‐SRC in a cohort of 18 patients, demonstrating the feasibility and perioperative safety, and provide a discussion of our results together with the relevant data in the literature.

## 2 MATERIALS AND METHODS

The prospectively collected surgical database of two highly experienced robotic urology surgeons (BT, PD) was searched for cases of RA‐SRC, defined as RARC performed post‐previous pelvic RT for palliative or oncologic treatment purposes. The demographic information collected included patient age at surgery, gender, body mass index (BMI), Charlson comorbidity index, indication for prior RT and indication for RC. Outcome measures were focused on the patients' operative course, hospital stay and complications. The Clavien–Dindo scale was used to grade postoperative complications.

### 2.1 Intervention

Robotic surgery was performed using a da Vinci surgical robot (Si and Xi model) and included RARC and urinary tract reconstruction. Unless previously excised, RC included removal of the bladder, prostate and seminal vesicles in men and the uterus, fallopian tubes and anterior vaginal wall in women. Pelvic lymph node dissection was performed when oncologically indicated. All patients received ileal conduit diversion. All cases were performed with a total intracorporeal approach.^8^

## 3 RESULTS

A total of 18 patients who underwent RA‐SRC between 2016 and 2023 were included in the current analysis. Patient's baseline characteristics are summarized in Table 1. As shown, the mean age was 74.3 years, mean BMI was 27.4 kg/m^2^ and mean Charlson score was 4.9. The most frequent indication for salvage cystectomy (*n* = 12) was palliation for refractory voiding dysfunction after prior pelvic RT and included complete urinary incontinence, recurrent bladder neck contracture, chronic radiation cystitis and intractable haematuria. The indication for surgery in the remaining six patients was oncological (MIBC or high risk NMIBC). All patients in the cohort had previous RT to the pelvis: *n* = 12 external beam RT (EBRT), *n* = 5 stereotactic body RT (SBRT) and *n* = 1 brachytherapy. Twelve patients also had prior radical pelvic surgery (*n* = 9 radical prostatectomy and *n* = 3 abdominoperineal resection). The median follow‐up period post‐surgery was 43.5 months. The operative and perioperative results are summarized in Table 2. The mean operative time was 262 min. Mean estimated blood loss (EBL) was 200 mL. One patient had one unit of blood transfusion intraoperatively due to low pre‐operative Hb level and 250 mL EBL during surgery. Median hospital length of stay (LOS) was 12.1 days (IQR 5–47 days). There were no intraoperative complications and no conversions to open surgery. There were eleven 90‐day postoperative complications (61.1%): three Clavien–Dindo grade 1, five Clavien–Dindo grade 2, two Clavien–Dindo grade 3b (one Pfannenstiel extraction wound dehiscence that required operative repair and one small bowel obstruction requiring laparotomy) and one Clavien–Dindo grade 4a (abdominal compartment syndrome with respiratory failure, renal failure, urine leak and sepsis). There was one late postoperative complication of left sided ureteroileal anastomotic stricture that required robotic‐assisted ureteric re‐implantation.

**TABLE 1:** Patient characteristics.

	Total
No. of patients	18
Age (years; mean ± SD)	74.3 ± 5.8
Sex (*n* [%])	
Male	16 (88.9)
Female	2 (11.1)
BMI kg/m^2^ (mean ± SD)	27.4 ± 3.9
Charlson score (mean)	4.9
Indication for RC (*n* [%])	
Palliation	12 (66.7)
Bladder cancer	6 (33.3)
Prior pelvic radiation (*n* [%])	
EBRT	12 (66.7)
SBRT	5 (27.8)
Brachytherapy	1 (5.6)
Indication for prior pelvic radiation (*n* [%])	
Prostate cancer	13 (72.2)
Bladder cancer	1 (5.6)
Rectal cancer	3 (16.7)
Endometrial cancer	1 (5.6)
Prior pelvic surgery (*n* [%])	12 (66.7)
Radical prostatectomy	9 (50)
Abdominoperineal resection	3 (16.7)
Type of urinary diversion (*n* [%])	
Ileal conduit	18 (100)
Lymph node dissection (*n* [%])	6 (33.3)

**TABLE 2:** Operative and perioperative results.

	Total
Operation time in min (mean ± SD)	262.5 ± 87.5
Intraoperative blood loss (mL) (mean ± SD)	200 ± 130.6
Intraoperative transfusion rate (*n* [%])	1 (5.5)
Conversion to open surgery (%)	0
90‐day postoperative complications (*n* [%])	11 (61.1)
Clavien ≥ III (*n* [%])	3 (16.7)
Postoperative length of stay in days (mean ± SD)	13.7 ± 12.1
Long‐term complications (*n* [%])	1 (5.6)

## 4 DISCUSSION

Prior pelvic RT results in tissue damage that is not limited to the organ of interest but can also affect surrounding pelvic organs including the bladder, rectum, urethra and small bowel. RT often results in a desmoplastic reaction, making identification and dissection of surgical planes difficult and results in a woody and indurated quality to affected tissue. Moreover, tissue ischaemia after radiation can lead to more vulnerable ureteric and intestinal tissue, with a higher risk of anastomotic leakage, prolonged ileus and delayed wound healing.^4^, ^5^ Previous pelvic surgery adds to the technical difficulty as it can result in obliteration of anatomical planes and higher rates of adhesions and fibrosis of tissues. Taken together, these factors make SRC a technically challenging operation, which may result in a higher risk of intra‐ and post‐operative complications. The risks versus benefits should be thoroughly evaluated, especially when surgery is contemplated for palliative purposes aimed at improving quality of life. The feasibility and safety of SRC has previously been reviewed mainly in the setting of open surgery. Eisenberg et al. reported the early complication rates in patients undergoing open SRC.^9^ In their retrospective series of 148 cases, the overall early complication rate (at 90 days) was 77%, with high‐grade complications in 32.4% of cases. The surgically‐related mortality rate at 90 days was 6.1%. Gastrointestinal complications were the most common, followed by infectious and bleeding complications. These postoperative complication rates are higher than the cumulative risk reported in cohorts of primary RC (i.e., without prior RT), which are 49% to 64% for overall complication rates and 13% to 30.8% for high‐grade complications with a related mortality rate of 2.7% to 5.8%.^10^, ^11^ Gontero et al. retrospectively analysed data from 682 patients who had undergone SRC at 25 high‐volume institutions.^12^ They showed that SRC is associated with a high risk of morbidity (75% risk of any complication and a 33% risk of a major complication) and a 90‐day mortality rate of 3.1%. In multivariable analysis adjusted for age, gender and type of RT, patients treated with RT for bladder cancer had 1.7 times increased relative risk of experiencing any complication after SRC compared to those with RT for prostate cancer (*p* = 0.023). Postoperative bleeding was the most common surgical complication (14%), followed by ureteroileal anastomotic strictures (9.2%) and bowel anastomosis leak (6.2%). Bowel leakage occurred predominantly during the early postoperative period, while ureteric strictures were late complications. Pieretti et al. compared SRC post‐TMT with SRC following pelvic RT for other indications and with primary RC.^13^ The study showed that all three are comparable in terms of intraoperative and early postoperative complication rates (60–76%), but SRC (either post‐TMT or following pelvic RT for other indications) was associated with a higher risk of major late complications (HR 2.3 and 2.1, respectively). The most common late complications included ureteroileal anastomotic strictures, small bowel obstruction and parastomal/ventral hernias. Anecdotally in the current series, the most technically challenging cases were those following prior radical prostatectomy with adjuvant pelvic radiation, but numbers were too small for a subgroup analysis.

Published data about RA‐SRC are very limited. Patel et al. retrospectively compared primary RARC with a small cohort of patients who underwent RARC following intervention for prostate cancer (10 patients post‐RT to the prostate and 19 postradical prostatectomy).^14^ They found no significant difference in perioperative outcomes between the groups, with the median EBL of 150–250 mL, median OT of 6.9–7.3 h and hospital LOS of 6–8.5 days (with a trend towards longer LOS in the post‐RT group). The 30‐day and 30‐ to 90‐day complication rates were not significantly different across the groups with rates of 60.53% and 71.71%, 73.68% and 84.21% and 60% and 70% in the primary, post‐RP and post‐RT RC groups, respectively. Elsayed et al. retrospectively evaluated the effect of complexity of the surgical field on perioperative outcomes of RARC.^15^ Complexity was defined as grade 1 for patients with a naïve pelvis, grade 2 for a single pelvic intervention (surgery or RT) and grade 3 for two or more pelvic interventions prior to RARC. Higher complexity grades exhibited higher 90‐day complication rates (grade 3: 74%, grade 2: 68% and grade 1 59%) and higher high‐grade complication rates (grade 3: 24%, grade 2: 18% and grade 1: 13%). On multivariate linear and logistic regression models, there was no significant difference between groups in blood loss, operative time and readmissions.

The early complication rate in our cohort corresponds to the data from Patel et al.^14^ and Elsayed et al.^15^ cohorts of SRC, with an overall complication rate of 61.1% and high‐grade complication rate of 16.7%. These rates appear to be lower than the complication rates reported in the open SRC cohorts (75–77% for any early complication and 32–33% for high‐grade complication) and is equivalent to the complication rates reported for open primary RC (49% to 64%).^10^, ^11^, ^12^ The long term complication rate in our cohort was only 5.6%, with only one patient developing ureteroileal anastomotic stricture that required surgical repair. Notably, two thirds of the patients in the current series would have been considered grade 3 according to Elsayed's classification.

Our data together with the limited published data from other cohorts of RA‐SRC suggest that in experienced hands, RA‐SRC is feasible, with intraoperative and perioperative complication rates that are lower than the published data for open SRC. This is supported by data from recent reviews showing fewer complication rates for RARC when compared with the open approach.^16^ Our study has several limitations. This is a retrospective analysis with a small cohort of patients. All cases in this cohort were performed by two highly experienced robotic surgeons, so these results may not be widely applicable. Further, only one patient in our cohort had RA‐SRC after failed TMT for MIBC; therefore, extrapolation of data from our cohort to this patient's population is limited, especially when previous data in the literature suggest that complication rates are higher for this population as compared to SRC postpelvic RT for other indication.^12^, ^13^ More studies are needed to establish the feasibility and safety of RA‐SRC. These data will assist with counselling patients that suffer from post‐radiation urinary symptoms about their treatment options and may also affect patients' decision making regarding their primary treatment for MIBC if proven to be reasonably safe post‐TMT.

## CONFLICT OF INTEREST STATEMENT

The authors declare that they have no known competing financial interests or personal relationships that could have appeared to influence the work reported in this article.
